# The adaptation of the desirability of outcome ranking for interventional clinical trials in epilepsy: A novel consumer‐led outcome measure

**DOI:** 10.1002/epi4.12839

**Published:** 2023-10-11

**Authors:** Lucy Vivash, Hannah Johns, Terence J. O'Brien, Leonid Churilov

**Affiliations:** ^1^ Department of Neuroscience Monash University Melbourne Victoria Australia; ^2^ Department of Neurology Alfred Health Melbourne Victoria Australia; ^3^ Department of Medicine (Royal Melbourne Hospital) University of Melbourne Melbourne Victoria Australia; ^4^ Department of Neurology Royal Melbourne Hospital Parkville Victoria Australia

**Keywords:** consumer design, epilepsy, interventional clinical trial, trial outcomes

## Abstract

Interventional clinical trials in epilepsy are typically designed and powered to detect a change in seizure frequency as the primary endpoint, with little consideration given to other benefits or harms of the therapy, or impacts on common epilepsy comorbidities. Desirability of outcome ranking (DOOR) is a novel methodology for evaluating benefits and harms associated with introduction of a new treatment. Multiple outcomes are combined and the resulting combinations are ranked according to their desirability. Herein we describe the adaptation of DOOR for use in therapy trials in epilepsy. Consumers with epilepsy were presented with a selection of measures typically included in epilepsy trials and asked to rank their importance in terms of a desirable outcome and to identify interactions between different seizure control levels and other measures. Seizure control, adverse events, and psychiatric comorbidities were identified as most important, and combinations of these outcomes were ranked to form epilepsy‐DOOR. A separate consumer discussion group verified the appropriateness and accuracy of the ranking. The resultant epilepsy‐DOOR includes 60 possible outcomes, representing high granularity for the assessment of future interventions. It demonstrates the importance of consumer involvement in trial design and presents an alternative to seizure frequency for evaluating new treatments for epilepsy.

## 1 INTRODUCTION

Clinical trials of new therapies for drug‐resistant epilepsy are typically powered to detect reductions in seizure frequency (most commonly >50% reduction) as the primary endpoint. However, additional factors, including adverse events (AEs), cognitive deficits, psychiatric comorbidities, quality of life and cost, are important to people with long‐standing epilepsy when considering the desirability of their epilepsy treatment.^1^, ^2^ To date almost all clinical therapeutic trials in epilepsy have been of symptomatic antiseizure medications (ASMs), but the development of disease‐modifying treatments has become a major focus of the translational epilepsy research effort,^3^ and this is likely to lead to future trials of disease‐modifying treatments. Having a broader assessment of the burden of epilepsy as a clinical trial endpoint is likely to be of particular relevance for future disease‐modifying treatment trials.

To better capture the multifaceted nature of epilepsy as a primary endpoint for clinical trials of treatment we sought to develop an *“epilepsy‐DOOR*”, an adaptation of The Desirability Of Outcome Ranking (DOOR)^4^ method. DOOR ranks the outcomes for participants according to the desirability of their overall outcome that may include a combination of benefits and harms. DOOR was initially developed for application to studies of inpatient antibiotic use, and the authors provide a comprehensive explanation of the principles and considerations when adapting DOOR for other uses.^4^

One advantage of DOOR over traditional approaches to measuring trial outcomes is that the overall outcome is constructed based on benefits and harms, synthesizing multiple measures for each individual patient. The possible outcomes are used to analyze the patient, rather than patients used to measure the outcome.^5^ The number and definition of categories is tailored to the disease of interest, with no set number of levels for each category. During analyses, the distributions of DOORs are compared between treatment arms.

The most important factor in the design of the DOOR is to ensure consensus of outcomes. Thus, we performed a two‐stage preference elicitation process with consumers to adapt the DOOR approach for use in epilepsy trials.

## 2 METHODS

### 2.1 Development of outcomes

**FIGURE 1:**
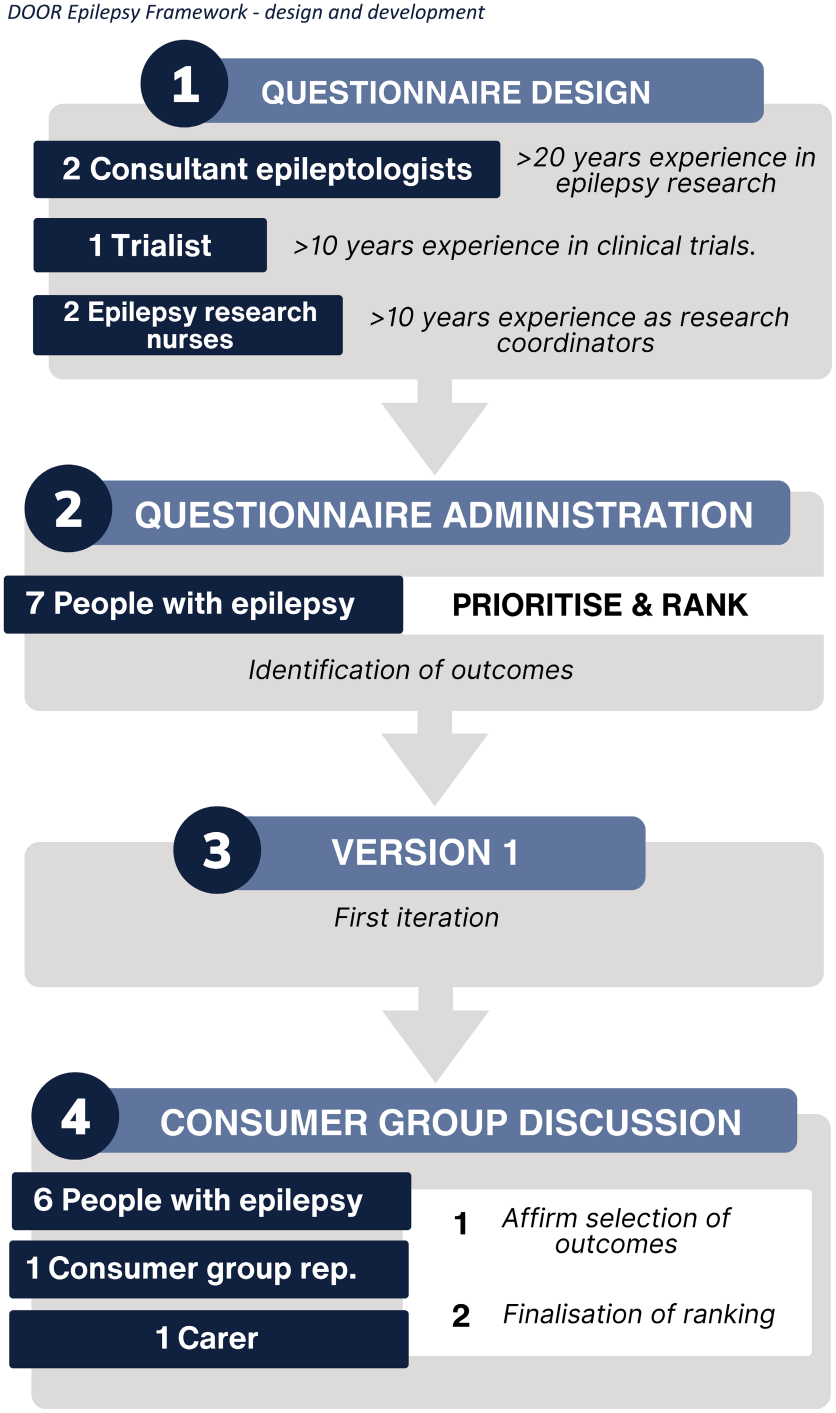
Schematic flow chart of the development of the epilepsy‐DOOR. The initial questionnaire was designed by experienced clinicians based on their clinical and research experience. The questionnaire was administered to people with epilepsy (*n* = 7), and the first version of the epilepsy‐DOOR constructed. This was then reviewed by the consumer focus group (*n* = 8) who revised and optimized the outcome selection and order of the rank.

Commonly measured outcomes in drug‐resistant epilepsy studies, that is, seizure frequency, AEs, cognitive symptoms, neuropsychiatric symptoms, ASM burden, were defined as measures for evaluation.^6^, ^7^, ^8^, ^9^ For each of these measures levels of outcome were defined by the authors based on their clinical experience (Table 1). Seven consumers with long‐standing drug‐resistant focal epilepsy (*n* = 5 female, Figure 1) were surveyed to identify and prioritize the top three outcomes for inclusion into the epilepsy‐DOOR. The consumers were asked to rank outcomes in order of importance, with additional questions on the interactions between outcomes to further define and order the DOOR. The first iteration of the epilepsy‐DOOR was constructed based on the responses to the survey.

**TABLE 1:** Potential outcomes for inclusion in DOOR‐epilepsy.

Seizure frequency*	Adverse events*	Quality of life*	Neuropsychiatric symptoms	Cognitive deficits	ASM burden
>90% reduction	No AEs	Improvement	Improvement	Improvement	Reduction/no change
>50% reduction	Mild temporary AEs	No change	No change	No change	Increase
>25% reduction	Mild ongoing AEs	Worsening	Worsening	Worsening	
<25% reduction	Moderate ongoing AEs				
	Severe ongoing AEs				

*Note*: Asterisks (*) indicate those included in the final epilepsy‐DOOR.

### 2.2 Consumer‐led outcome review

The first iteration of the epilepsy‐DOOR was reviewed and refined by a separate group of eight consumers (6 people with epilepsy, one consumer representative, and one carer of a person with epilepsy, female *n* = 6). In accordance with the Cancer Australia Consumer Involvement Toolkit and the Australian Clinical Trials Alliance (ACTA) Consumer Involvement and Engagement Toolkit,^10^ consumers were sought through expressions of interest on epilepsy consumer groups (Epilepsy Foundation of Victoria, Epilepsy Action Australia, Australian Women with Epilepsy) social media channels. Respondents to the expression of interest were contacted to further explain the purpose of the consumer group and to affirm participation. The consumer group discussion occurred over Zoom to facilitate participation from people across Australia. As per the ACTA Consumer Involvement and Engagement Toolkit ethical approval was not required. Except that described above, no demographic or clinical information was collected to prevent potential identification of consumers.

The group discussion followed the outline provided by the ACTA toolkit and included a brief outline of the proposed clinical trial and a description of the surveys conducted previously. The main discussion centered around the epilepsy‐DOOR as constructed from the surveys. The discussion was an open forum with a free‐flowing discussion between the consumers, considering the measures included in the DOOR, suggesting alternatives, and reordering the ranking.

## 3 RESULTS

### 3.1 Category definitions

The consumer surveys resulted in prioritization of a reduction in seizure frequency, followed by AEs, psychiatric symptoms, ASM burden, and cognitive symptoms. The top three were subsequently entered into the epilepsy‐DOOR.

Outcomes are measured longitudinally comparing baseline measures to the follow‐up measure(s) as available. Change in seizure frequency is defined as >90% reduction, >50% reduction, >25% reduction, or <25% reduction as recorded in participant seizure diaries. AEs are defined according to the CTCAE gradings, with AEs of any severity lasting <72 h considered mild temporary for the purposes of the DOOR. Change in quality of life scores are measured using QOLIE‐31, with a change of ±5.19 or more being an improvement/worsening of quality of life.^11^ Neuropsychiatric symptoms are measured using the NDDI‐E and GAD‐7, with changes of ≥6 and ≥4, respectively, indicative of change in symptom severity. Change in cognitive performance is defined as ±3 points on the verbal paired associates delay (A7).^12^ Increase in ASM burden is defined as introduction of a new ASM or a ≥25% increase in dose of a current ASM.

### 3.2 Category inclusion and prioritization

The consumer prioritization of levels of outcomes, resulting in a ranking of 60 possible individual ranks: the full factorial combination of four levels on seizure frequency, five levels on AEs, and three levels on neuropsychiatric symptoms. The first iteration of the DOOR‐epilepsy is found in Table 2. Briefly, the DOOR can be split into four major strata: the bottom stratum, with the worst outcomes (49–60) characterized by severe ongoing AEs, irrespective of effects on other measures, the third stratum represented by minimal (<25%) reduction in seizure frequency (37–48); the second stratum (28–36), again represented by moderate ongoing AEs, and the first stratum (1–27), which is not represented by a singular factor.

**TABLE 2:** The full epilepsy‐DOOR prior to and following the consumer discussion forum.

Strata	
First (1‐27)	Third (37‐48)
Second (28‐36)	Fourth (49‐60)

Epilepsy‐DOOR	Original DOOR			Post‐consumer forum DOOR		
	Reduction in seizure frequency	Adverse events	Change in psychiatric	Reduction in seizure frequency	Adverse events	Change in quality of life*
1	>90%	Nil	Improvement	>90%	Nil	Improvement
2	>90%	Nil	No change	>90%	Nil	No change
3	>90%	Mild temporary	Improvement	>90%	Mild temporary	Improvement
4	>90%	Mild temporary	No change	>90%	Mild temporary	No change
5	>90%	Mild ongoing	Improvement	>90%	Mild ongoing	Improvement
6	>90%	Mild ongoing	No change	>90%	Mild ongoing	No change
7	>50%	Nil	Improvement	>50%	Nil	Improvement
8	>50%	Nil	No change	>50%	Nil	No change
9	>90%	Nil	Worsening	>90%	Nil	Worsening
10	>90%	Mild temporary	Worsening	>90%	Mild temporary	Worsening
11	>90%	Mild ongoing	Worsening	>90%	Mild ongoing	Worsening
12*	>50%	Nil	Worsening	>50%	Mild* temporary	Improvement*
13*	>50%	Mild temporary	Improvement	>50%	Mild temporary	No change*
14*	>50%	Mild temporary	No change	>50%	Nil*	Worsening*
15	>50%	Mild temporary	Worsening	>50%	Mild temporary	Worsening
16	>25%	Nil	Improvement	>25%	Nil	Improvement
17	>25%	Nil	No change	>25%	Nil	No change
18*	>25%	Nil	Worsening	>25%	Mild temporary*	Improvement*
19*	>25%	Mild temporary	Improvement	>25%	Mild temporary	No change*
20*	>25%	Mild temporary	No change	>25%	Nil*	Worsening*
21*	>25%	Mild temporary	Worsening	>50%*	Mild ongoing*	Improvement*
22*	>50%	Mild ongoing	Improvement	>25%*	Mild temporary*	Worsening*
23	>50%	Mild ongoing	No change	>50%	Mild ongoing	No change
24	>50%	Mild ongoing	Worsening	>50%	Mild ongoing	Worsening
25	>25%	Mild ongoing	Improvement	>25%	Mild ongoing	Improvement
26	>25%	Mild ongoing	No change	>25%	Mild ongoing	No change
27	>25%	Mild ongoing	Worsening	>25%	Mild ongoing	Worsening
28	>90%	Moderate ongoing	Improvement	>90%	Moderate ongoing	Improvement
29	>90%	Moderate ongoing	No change	>90%	Moderate ongoing	No change
30	>90%	Moderate ongoing	Worsening	>90%	Moderate ongoing	Worsening
31	>50%	Moderate ongoing	Improvement	>50%	Moderate ongoing	Improvement
32	>50%	Moderate ongoing	No change	>50%	Moderate ongoing	No change
33	>50%	Moderate ongoing	Worsening	>50%	Moderate ongoing	Worsening
34	>25%	Moderate ongoing	Improvement	>25%	Moderate ongoing	Improvement
35	>25%	Moderate ongoing	No change	>25%	Moderate ongoing	No change
36	>25%	Moderate ongoing	Worsening	>25%	Moderate ongoing	Worsening
37	<25%	Nil	Improvement	<25%	Nil	Improvement
38	<25%	Nil	No change	<25%	Nil	No change
39	<25%	Nil	Worsening	<25%	Nil	Worsening
40	<25%	Mild temporary	Improvement	<25%	Mild temporary	Improvement
41	<25%	Mild temporary	No change	<25%	Mild temporary	No change
42	<25%	Mild temporary	Worsening	<25%	Mild temporary	Worsening
43	<25%	Mild ongoing	Improvement	<25%	Mild ongoing	Improvement
44	<25%	Mild ongoing	No change	<25%	Mild ongoing	No change
45	<25%	Mild ongoing	Worsening	<25%	Mild ongoing	Worsening
46	<25%	Moderate ongoing	Improvement	<25%	Moderate ongoing	Improvement
47	<25%	Moderate ongoing	No change	<25%	Moderate ongoing	No change
48	<25%	Moderate ongoing	Worsening	<25%	Moderate ongoing	Worsening
49	>90%	Severe ongoing	Improvement	>90%	Severe ongoing	Improvement
50	>90%	Severe ongoing	No change	>90%	Severe ongoing	No change
51	>90%	Severe ongoing	Worsening	>90%	Severe ongoing	Worsening
52	>50%	Severe ongoing	Improvement	>50%	Severe ongoing	Improvement
53	>50%	Severe ongoing	No change	>50%	Severe ongoing	No change
54	>50%	Severe ongoing	Worsening	>50%	Severe ongoing	Worsening
55	>25%	Severe ongoing	Improvement	>25%	Severe ongoing	Improvement
56	>25%	Severe ongoing	No change	>25%	Severe ongoing	No change
57	>25%	Severe ongoing	Worsening	>25%	Severe ongoing	Worsening
58	<25%	Severe ongoing	Improvement	<25%	Severe ongoing	Improvement
59	<25%	Severe ongoing	No change	<25%	Severe ongoing	No change
60	<25%	Severe ongoing	Worsening	<25%	Severe ongoing	Worsening

*Note*: Four strata are clearly identified (noted by different colors). Changes made after the consumer discussion are highlighted by italics and asterisks (*). The worst outcomes (49–60, blue) are dominated by severe ongoing adverse events, irrespective of seizure control or changes to quality of life. The third stratum (37–48, orange) is identified by minimal reduction in seizure frequency, and the second stratum (28–36, yellow) by moderate ongoing adverse events, again irrespective of seizure control. The first stratum (1–27, green) is characterized by minimal ongoing adverse events, with greater reductions in seizure frequency and benefits to quality of life having the highest rank.

The consensus from the consumer group discussion was that quality of life (*which was not included as an outcome measure in the original questionnaires*) was more important than neuropsychiatric symptoms and thus a quality of life questionnaire (QOLIE‐31) was included as the third outcome in the epilepsy‐DOOR. The consumer discussion did not result in any changes to the bottom 3 strata of rankings; however, numerous minor changes were made to the first stratum as shown in Table 2.

### 3.3 Application/implementation of epilepsy‐DOOR to compare a random pair of participants

To enable the analysis within the win odds framework (see below for details) it is necessary to compare all participants as randomly selected pairs allocated to different study arms. The following example demonstrates the application of epilepsy‐DOOR in a hypothetical clinical trial. At baseline, Patient A has 1 seizure per day and scores 72 on the QOLIE‐31. They are randomized to the active treatment arm and commence the study drug. They experienced mild nausea and dizziness in the first 2 days, but this was resolved by day 3 (*mild temporary AE*). They report no other AEs. At the end of the study, their seizures have reduced to 1 every 3 days (*>50% reduction*), and their QOLIE‐31 score is 78 (+6 points; *improvement*). As defined in epilepsy‐DOOR tool, Patient A's epilepsy‐DOOR is 12. Patient B is randomized to the placebo arm. They have 5 seizures per month and a QOLIE‐31 score of 79 at baseline. They do not report any AEs during the study (*nil AEs*). In the end, their seizures have reduced to 3 per month (*>25% reduction*) and their QOLIE‐31 score is 80 (+1 point; *no change*), thus their epilepsy‐DOOR is 17. Comparing these two patients would result in a “win” for the treatment arm and a “loss” for the placebo arm as Patient A's rank is better than Patient B's. Patient C is randomized to the treatment arm. At baseline, they have 4 seizures per week and a QOLIE‐31 score of 65. They experience intermittent moderate dizziness and somnolence while taking the study medication (*moderate AE*). At the end of the study, their seizures are reduced to 1 per week (*>50% reduction*), and their QOLIE‐31 score is 67 (+2 points; *no change*). Patient C's epilepsy‐DOOR is 32. The comparison between Patient B and Patient C will result in a “loss” for the treatment arm and a “win” for the placebo arm. Patient D is randomized to the placebo arm. They have 1 seizure per week and a QOLIE‐31 score of 68 at baseline. They do not report any AEs (*nil AEs*). At the end of the study, their seizure frequency remains 1 per week (*<25% reduction*) and their QOLIE‐31 score is 75 (+7 point; *improvement*), resulting in an epilepsy‐DOOR of 37. Patient C is ranked higher than Patient D, resulting in a “win” for the treatment arm and a “loss” for the placebo arm.

Using the examples described above, if Patients A and B and Patients C and D were compared, Patients A and C would both “win,” indicating superiority (or greater benefit than harm) of the treatment over placebo. However, if Patients A and D, and Patients B and C were compared, Patients A and B would “win” demonstrating equal wins between the two groups and, therefore, no benefit of treatment.

### 3.4 Utilization of epilepsy‐DOOR as a study outcome

During the trial outcome analyses, the DOOR distributions are compared between trial arms. Confidence intervals are used to estimate the treatment effect that tallies all the recorded “wins” and “losses” from all possible treatment/placebo patient pairs into the probability (or the odds) that a randomly selected participant in arm A will have a better DOOR than a randomly selected participant in arm B. In case “ties” are recorded, their number is split equally between the two arms. If there is no difference between the arms then such probability will be ~50% (odds ~1), while greater differences in DOOR distributions will result in probability (odds) values away from that value. Sample size estimations are based on a superiority test, the null hypothesis being that there is no difference in the DOOR between arms, and the alternate being that arm A has a higher DOOR (i.e., the probability that a randomly selected participant will have a higher DOOR if assigned to arm A differs from 50% [odds differ from 1]).

The Win Odds^13^ is calculated using the following formula:

$$\mathrm{Win}\;\text{Odds}=\frac{\left(\text{wins}+\text{ties}/2\right)}{\left(\text{losses}+\text{ties}/2\right)}$$

Sample size calculations can be completed based on the Win Odds, using estimated proportions of “wins,” “ties,” and “losses” between groups, see Johns et al.^14^ for further details and a downloadable tool on calculating sample sizes from Win Odds.

## 4 DISCUSSION

Here we report a novel consumer‐codesigned trial outcome measure, *epilepsy‐DOOR*, designed by adapting the previously reported DOOR approach for use in adult drug‐resistant epilepsy clinical trials. Epilepsy‐DOOR combines three common epilepsy trial outcome measures and integrates them into a single outcome measure. This diversifies clinical trial methodologies, extending co‐primary endpoints into a multifaceted, but single measure of study outcome. Interventional epilepsy studies are typically powered to detect a >50% reduction in seizure frequency for adjunctive therapies in drug‐resistant epilepsy and seizure freedom rates in newly diagnosed epilepsy. Neither of these approaches reflects treatment‐emergent AEs or other potential harms that could impact long‐term treatment compliance. We developed epilepsy‐DOOR for use as the primary outcome in our forthcoming clinical trial of sodium selenate as a disease‐modifying treatment for epilepsy (Vivash et al., *in press*, ACTRN12623000446662). In this trial, we will use ‘standard’ outcome measures (seizure frequency, quality of life, cognition, neuropsychiatric symptoms) and statistical analysis techniques (LMEMs) as secondary outcomes. By utilizing these established techniques as secondary measures, we will evaluate the epilepsy‐DOOR framework against the standard methods. How epilepsy‐DOOR is received by the regulatory authorities remains to be seen. However, we believe that it represents a relevant outcome measure for disease‐modifying treatment trials, rather than the traditional symptomatic antiseizure medication trials, as it aligns with the increasing understanding that the burden of epilepsy represents more than seizure frequency alone.^1^, ^2^, ^15^ The FDA patient‐reported outcome measures (PROMs) guidance document,^16^ provides a framework for development, validation, and use of PROMs as trial endpoints. DOORs could be validated through a similar pathway. Application to real‐world data will be the first step in the evaluation and optimization of epilepsy‐DOOR as an outcome measure for future pivotal trials.

The epilepsy‐DOOR includes measures that have some degree of overlap. The QOLIE‐31 includes questions about seizure worry and perception of some common adverse events, which will be influenced by seizure burden and presence of adverse events, respectively. While these will be somewhat interdependent, there are subtle differences in the capture and characterization of these measures (presence vs perception of adverse events, quantification vs perception of seizures) and the time frame for information (52 weeks for the adverse events, previous 4 weeks for QOLIE‐31). The QOLIE‐31 is also heavily impacted by mood, therefore, when using the epilepsy‐DOOR it will be necessary to ensure maintenance of mood stabilizers to demonstrate the impacts on the QOLIE‐31 (and, therefore, epilepsy‐DOOR) are driven by changes to someone's epilepsy and downstream impacts on mood rather than changes in mood symptoms driven by response to mood stabilizers independent of epilepsy treatment.

While the epilepsy‐DOOR measure reported here is only applicable to interventional studies in long‐standing drug‐resistant focal epilepsy in adults with relatively mild comorbidities, the consumer population with whom it was developed, we have demonstrated the adaptation of the DOOR framework to a chronic neurological disease. Similar consumer‐driven multioutcome measures could be tailored to pediatric populations, or other developmental and epileptic encephalopathies as well as other diseases with relative ease. Evans et al.^4^ provide key considerations for adaptation and a use case example.

## AUTHOR CONTRIBUTIONS

LV and LC conceived of the study. All authors contributed to study design. LV wrote the manuscript. All authors reviewed and approved the final version.

## CONFLICT OF INTEREST STATEMENT

The authors report no conflicts of interest in relation to the present work.

## ETHICS STATEMENT

We confirm that we have read the Journal's position on issues involved in ethical publication and affirm that this report is consistent with those guidelines.
